# Supplementary material for: Angioplasty induces epigenomic remodeling in injured arteries
Source: Life Sci Alliance. 2022 Feb 15;5(5):e202101114. doi: 10.26508/lsa.202101114 (PMC8860099; doi:10.26508/lsa.202101114)
Supplement: Supplementary file 1 [file LSA-2021-01114_TableS1.docx]

**Supplemental Tables**

**Table S1. Mean coverage of ChIPseq peaks**

| **ChIP** | ***Artery Sample*** | ***Ccnd1*** | ***Uhrf1*** | ***Nrp2*** | ***Ezh2-Box1*** | ***Ezh2-Box2*** | ***Cdkn1c*** | ***Bmp4*** |
| --- | --- | --- | --- | --- | --- | --- | --- | --- |
| BRD4 | Injured | 11.4 | 5.0 | 8.3 | 12.5 | 18.3 | 9.1 | 3.7 |
|  | Uninjured | 8.6 | 3.8 | 6.1 | 5.0 | 13.5 | 13.8 | 9.0 |
| H3K27ac | Injured | 25.7 | 9.6 | 19.7 | 20.9 | 81.2 | 20.8 | 5.7 |
|  | Uninjured | 10.4 | 3.0 | 9.8 | 7.4 | 28.4 | 12.1 | 17.3 |
| H3K27me3 | Injured | 4.5 | 2.0 | 2.8 | 1.7 | 0.0 | 46.9 | 19.1 |
|  | Uninjured | 9.2 | 3.6 | 3.1 | 3.9 | 1.3 | 6.9 | 4.5 |
| H3K4me1 | Injured | 14.7 | 8.0 | 11.0 | 6.7 | 3.4 | 19.4 | 5.4 |
|  | Uninjured | 15.1 | 6.5 | 8.5 | 4.0 | 5.1 | 21.9 | 10.3 |

Note: The gene intervals querried include the regions shown on the IGV graphs.
