# Supplementary material for: Angioplasty induces epigenomic remodeling in injured arteries
Source: Life Sci Alliance. 2022 Feb 15;5(5):e202101114. doi: 10.26508/lsa.202101114 (PMC8860099; doi:10.26508/lsa.202101114)

F4-C

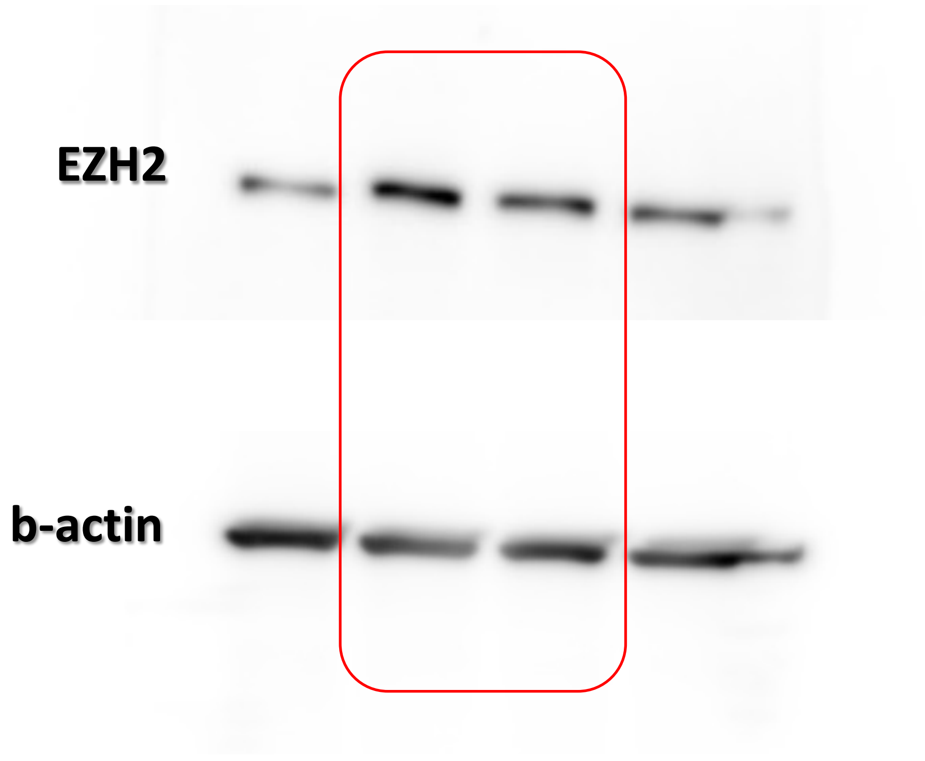

F4-E

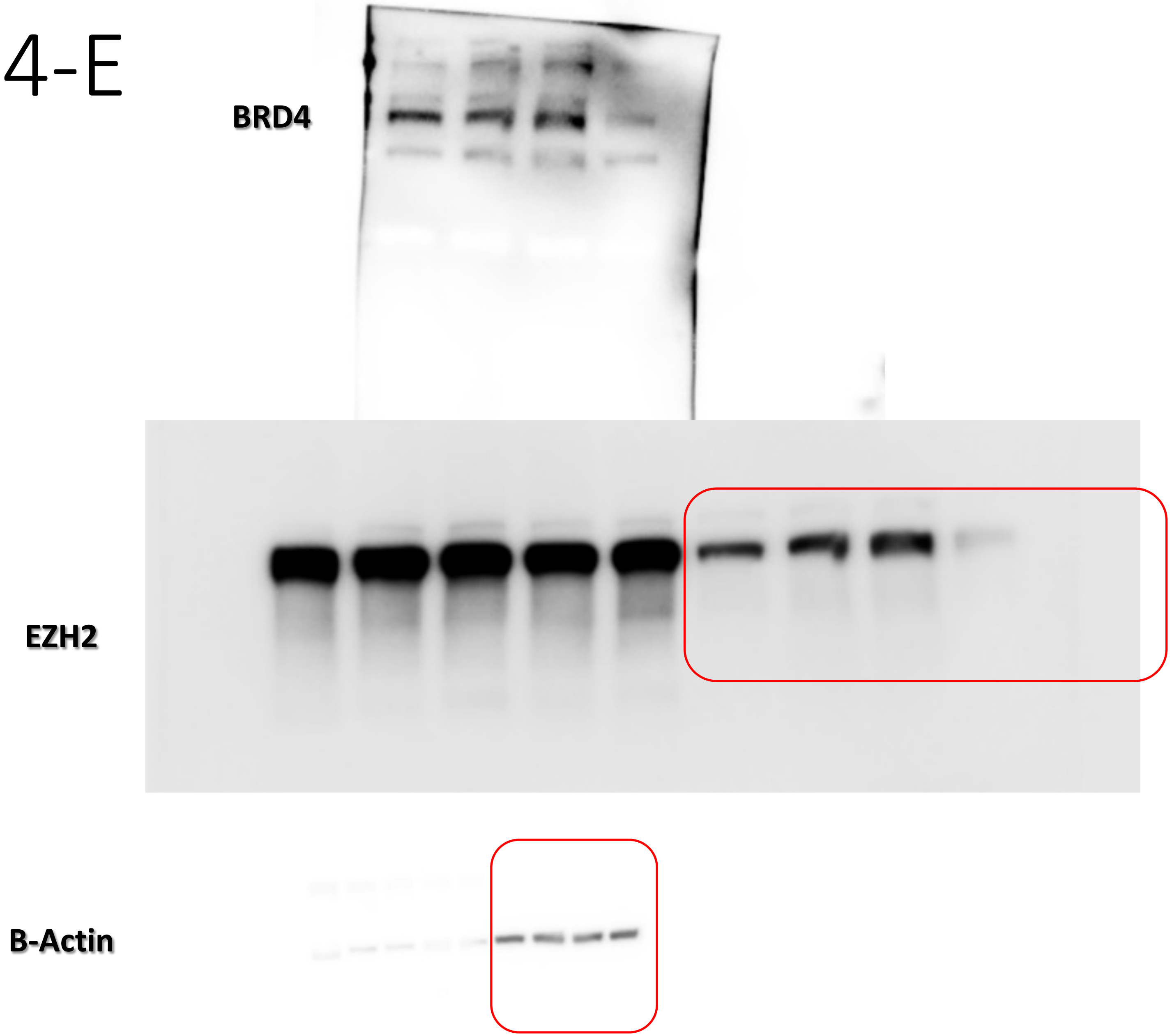

F8-A

EZH1

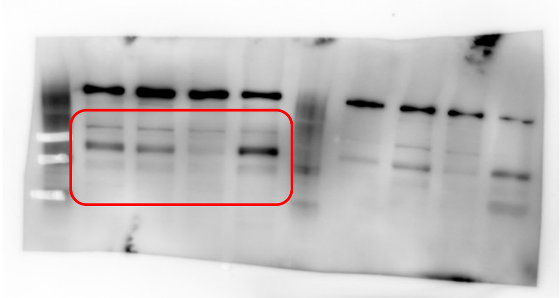

Actin

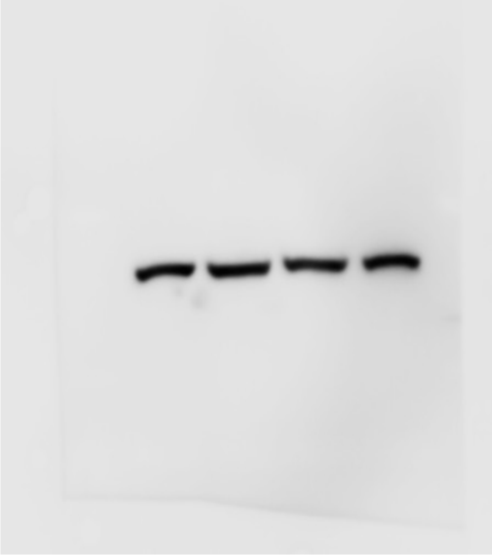

EZH2

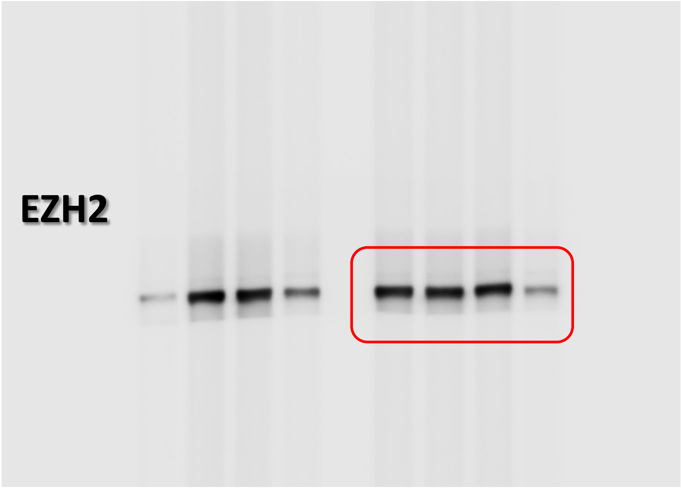

H3K27me3

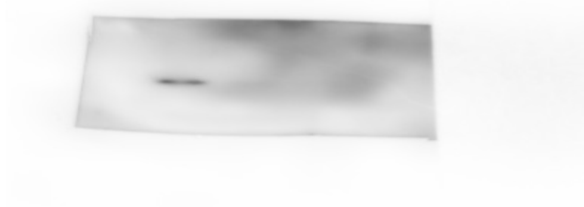

F8-E

EZH1

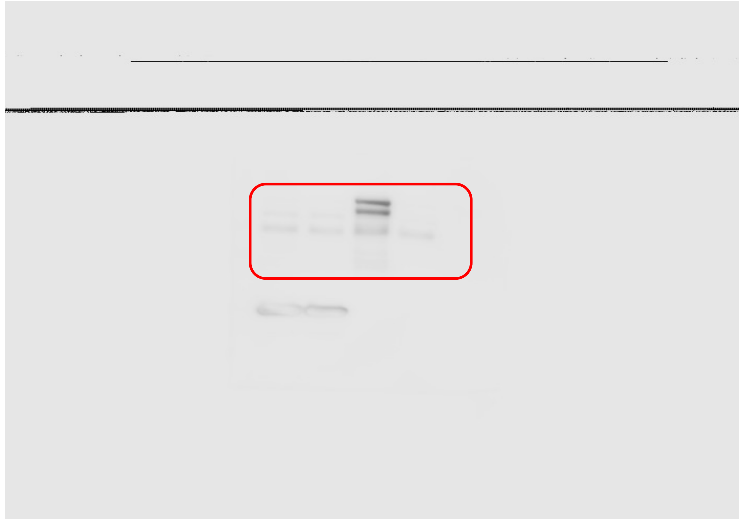

Actin

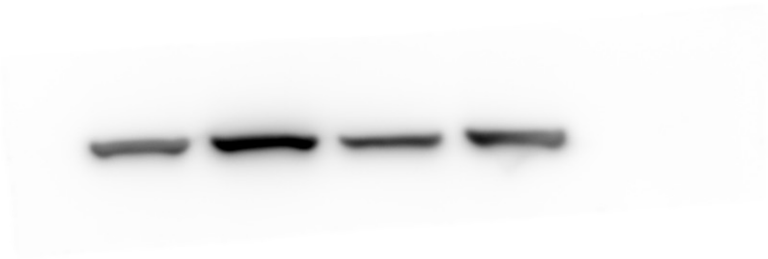

EZH2

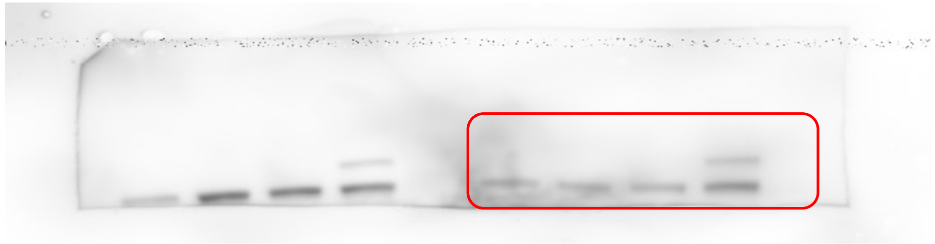

H3K27me3

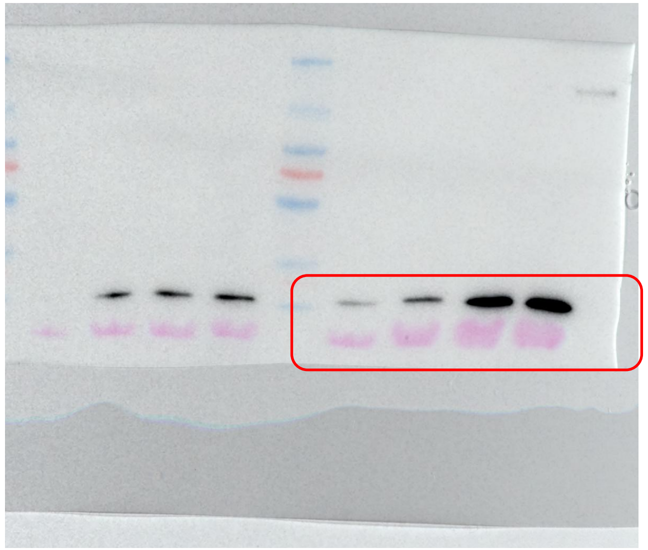

F9-A

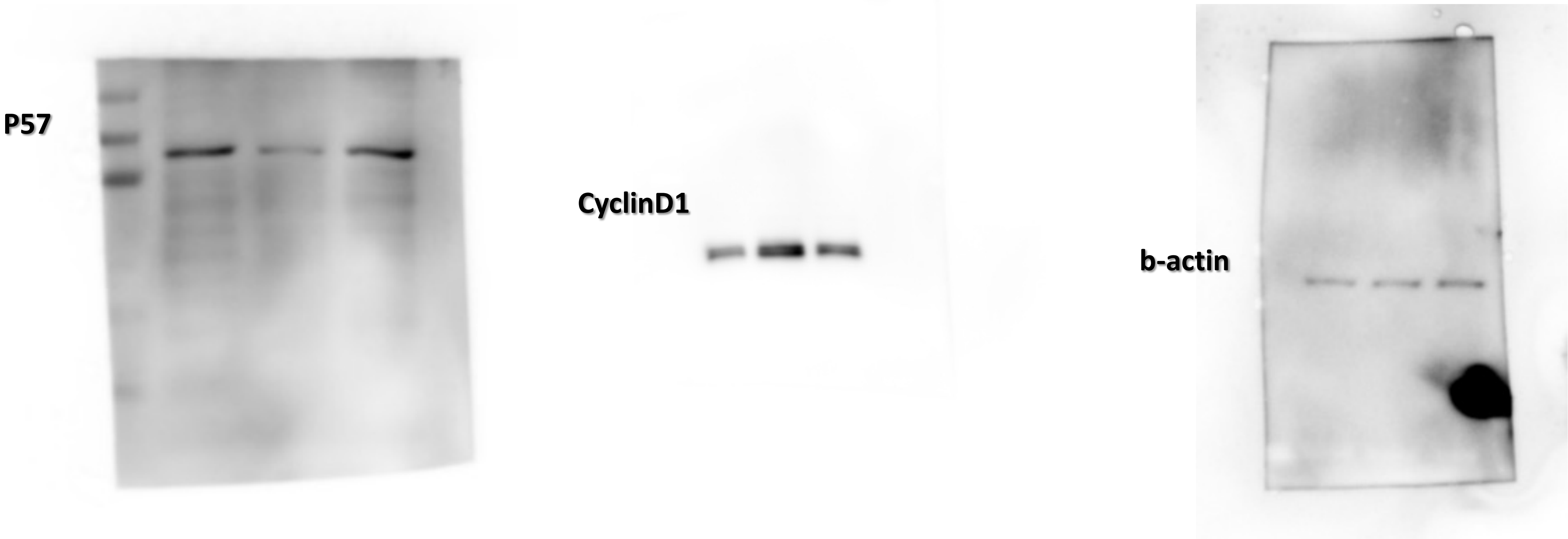

F9-D

P57

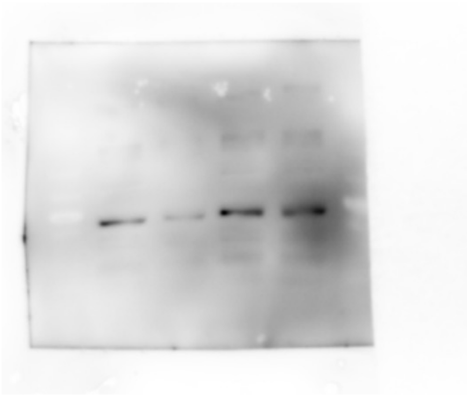

CyclinD1

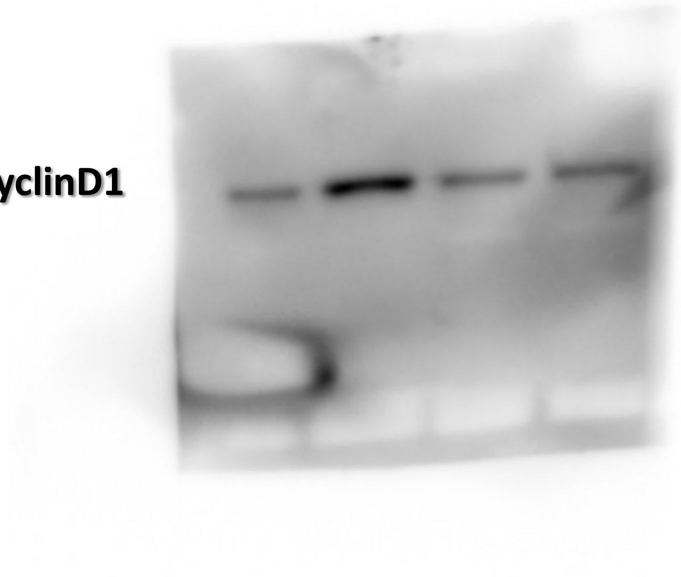

b-actin

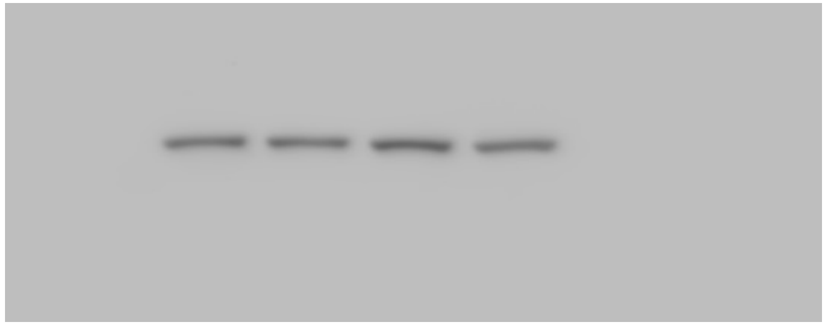

F9-G

P57

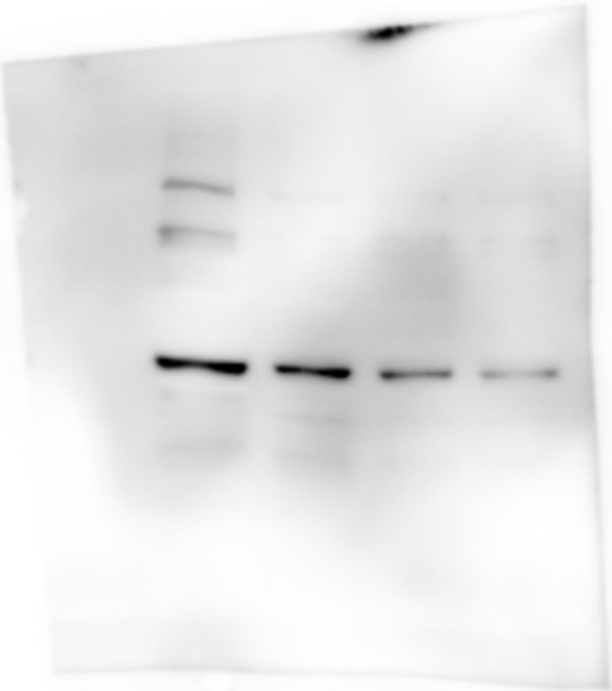

b-actin

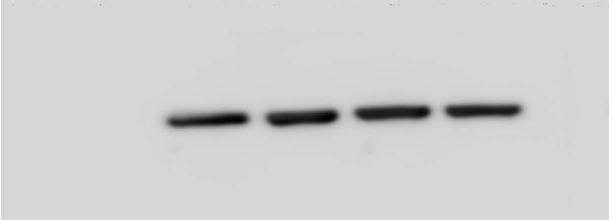

CyclinD1

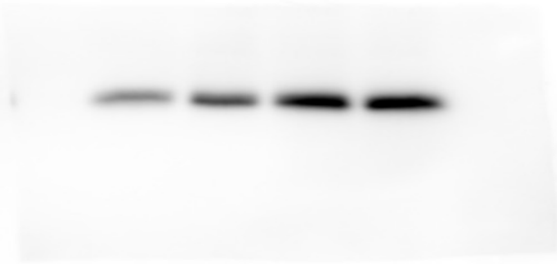

FigS6B

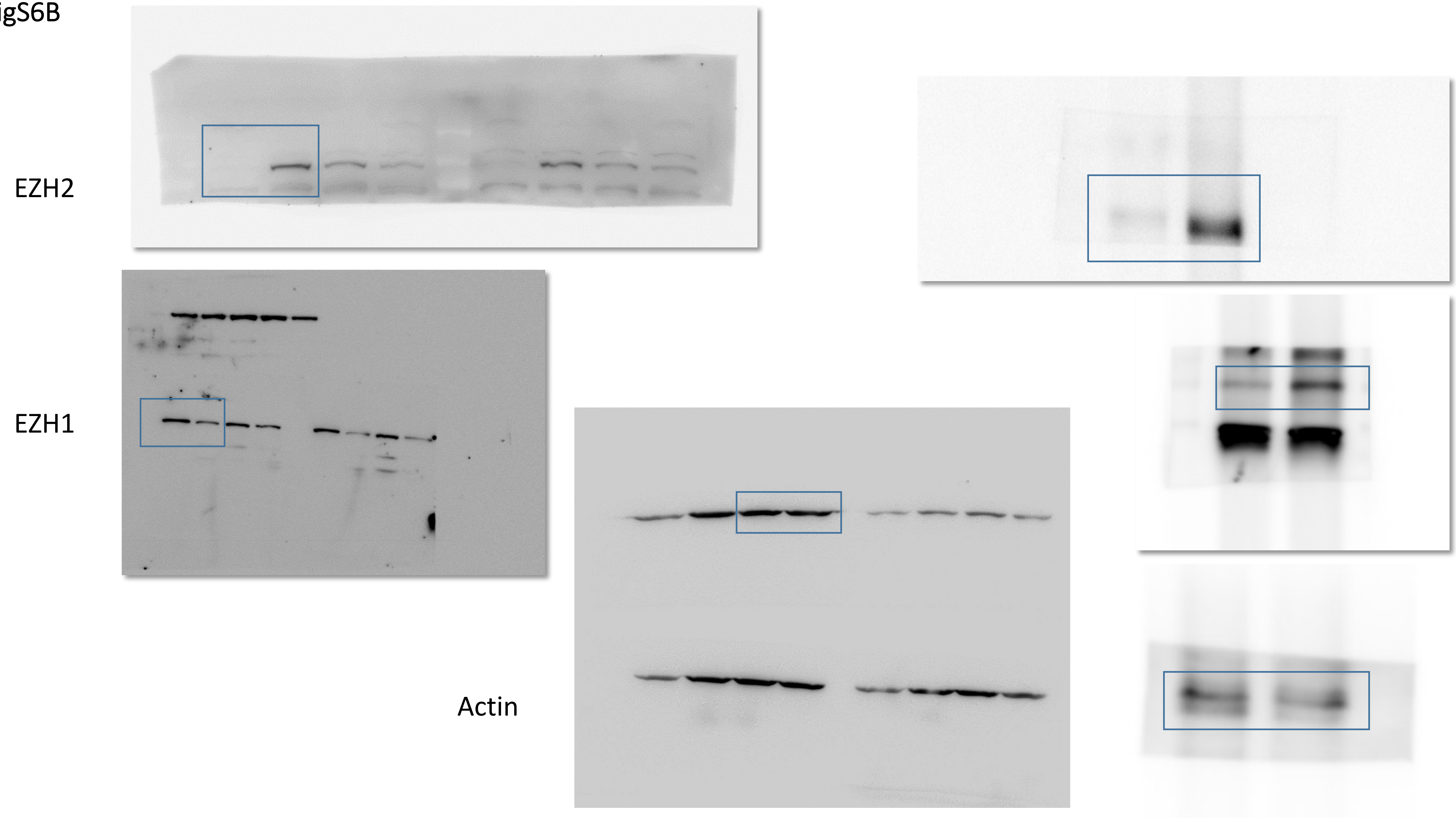

Supplement: Supplementary file 2 [file LSA-2021-01114_SdataF4_F8_F9_FS6.pdf]
