# Supplementary material for: Angioplasty induces epigenomic remodeling in injured arteries
Source: Life Sci Alliance. 2022 Feb 15;5(5):e202101114. doi: 10.26508/lsa.202101114 (PMC8860099; doi:10.26508/lsa.202101114)
Supplement: Supplementary file 3 [file LSA-2021-01114_TableS2.docx]

**Supplemental Tables**

**Table S2. Primers for Brd4^-/-^ genotyping**

| Mouse BRD4 flox | Forward: CCTGTGTGCACTTGCTCCCGAGGAGAGA |
| --- | --- |
|  | Reverse: GGACTAGAAACCTCCCAAATGTCTACAA |
| Mouse MYH11 cre/ERT2 | Forward: TGACCCCATCTCTTCACTCC |
|  | Reverse: AGTCCCTCACATCCTCAGGTT |
