# Supplementary material for: Angioplasty induces epigenomic remodeling in injured arteries
Source: Life Sci Alliance. 2022 Feb 15;5(5):e202101114. doi: 10.26508/lsa.202101114 (PMC8860099; doi:10.26508/lsa.202101114)
Supplement: Supplementary file 4 [file LSA-2021-01114_TableS3.docx]

**Supplemental Tables**

**Table S3. siRNA or shRNA sequences for mouse and rat genes**

| mouse EZH1 | sense: CATCGAAGAGCTGATCAATAA |
| --- | --- |
|  | Anti-sense: TTATTGATCAGCTCTTCGATG |
| mouse EZH2 | sense: GCACAAGTCATCCCGTTAAAG |
|  | Anti-sense: CTTTAACGGGATGACTTGTGC |
| rat BRD2 | sense: GCUUGAACGAUACGUUUUA |
|  | Anti-sense: UAAAACGUAUCGUUCAAGC |
| rat BRD3 | sense: AGGAAACCAUUGUCAACAATT |
|  | Anti-sense: UUGUUGACAAUGGUUUCCUCT |
| rat BRD4 | sense: GCAUCAACUUCUCCGCAGATT |
|  | Anti-sense: UCUGCGGAGAAGUUGAUGCTT |
