# Supplementary material for: Angioplasty induces epigenomic remodeling in injured arteries
Source: Life Sci Alliance. 2022 Feb 15;5(5):e202101114. doi: 10.26508/lsa.202101114 (PMC8860099; doi:10.26508/lsa.202101114)
Supplement: Supplementary file 5 [file LSA-2021-01114_TableS4.docx]

**Supplemental Tables**

**Table S4**. **Antibodies**

| **Antigen** | **Manufacturer** | **Catalog Number** | **Dilution Ratio** | **Application** |
| --- | --- | --- | --- | --- |
| H3K27ac | Active Motif | 39133 |  | ChIP |
| H3K4me1 | Active Motif | 61633 |  | ChIP |
| H3K27me3 | Cell Signaling Technology | 9733 | 1:1000 | Western Blot |
| H3K27me3 | Cell Signaling Technology | 9733 | 1:100 | Immunofluorescence |
| BRD4 | Abcam | Ab128874 | 1:1000 | Western Blot |
| BRD4 | LSbio | C804367 | 1:100 | Immunofluorescence |
| EZH1 | Proteintech | 20852-1-AP | 1:500 | Western Blot |
| EZH2 | Cell Signaling Technology | 5246 | 1:1000 | Western Blot |
| EZH2 | LSbio | B7096 | 1:100 | Immunofluorescence |
| UHRF1 | LSbio | C806412 | 1:100 | Immunofluorescence |
| PCNA | Abcam | Ab29 | 1:100 | Immunofluorescence |
| CyclinD1 | Cell Signaling Technology | 55506 | 1:1000 | Western Blot |
| P57 | Cell Signaling Technology | 2557 | 1:1000 | Western Blot |
| β-actin | Abcam | Ab8226 | 1:5000 | Western Blot |
