# Supplementary material for: Angioplasty induces epigenomic remodeling in injured arteries
Source: Life Sci Alliance. 2022 Feb 15;5(5):e202101114. doi: 10.26508/lsa.202101114 (PMC8860099; doi:10.26508/lsa.202101114)
Supplement: Supplementary file 6 [file LSA-2021-01114_TableS5.docx]

**Supplemental Tables**

**Table S5. Primer sequences for mouse and rat genes (qRT-PCR)**

| rat GAPDH | Forward: AAGGTCGGTGTGAACGGATTT |
| --- | --- |
|  | Reverse: CTTTGTCACAAGAGAAGGCAGC |
| rat BRD2 | Forward: GGGTCTGCCGGATTATCACA |
|  | Reverse: GCCCCCTTCTTATGGCTGTT |
| rat BRD3 | Forward: AAGATGGTGAGGTCCCACAG |
|  | Reverse: GGTACTCACGGCTGTCCATT |
| rat BRD4 | Forward: CTGCCAGTAATGGGGGATGG |
|  | Reverse: ATTGGTGCTGGCTGCATTTG |
| rat EZH1 | Forward: CTACCAACCCTGTGACCACC |
|  | Reverse: ACTCACGAACGGCCAAGTAG |
| rat EZH2 | Forward: TGTGAGCTCATTACGCGG |
|  | Reverse: GGGTGTTGCATGAAAGGGATG |
| mouse EZH1 | Forward: AGTGCTTCCTGCTCCAATGC |
|  | Reverse: TCACACTCACGAACTGCCAA |
| mouse EZH2 | Forward: CGCCTCGGTGCCTATAATGT |
|  | Reverse: GTGCTGGGTCTGCTACTGTT |
| mouse GAPDH | Forward: GAGAGTGTTTCCTCGTCCCG |
|  | Reverse: ATGGGCTTCCCGTTGATGAC |
| mouse P57 | Forward: CATGTCCGACGTGTACCTCC |
|  | Reverse: GTTCTCCTGCGCAGTTCTCT |
| mouse UHRF1 | Forward: AGACCTCTCTGGCAACAAGC |
|  | Reverse: CATCATAGCGGTTGCCCTCT |
| mouse cyclinD1 | Forward: GCAAGCATGCACAGACCTTT |
|  | Reverse: GGTCCTTGTTTAGCCAGAGGC |
