# Supplementary material for: Angioplasty induces epigenomic remodeling in injured arteries
Source: Life Sci Alliance. 2022 Feb 15;5(5):e202101114. doi: 10.26508/lsa.202101114 (PMC8860099; doi:10.26508/lsa.202101114)
Supplement: Supplementary file 7 [file LSA-2021-01114_TableS6.docx]

**Supplemental Tables**

**Table S6. Primer sequences for ChIP-qPCR**

| Rat EZH2 | Forward: TGGTAACGGTCTTAACCGCC |
| --- | --- |
|  | Reverse: CTTTCTGTCCGTGTCGCCAA |
| Rat UHRF1 | Forward: TTTTCTGATGTGACCGCGTG |
|  | Reverse: GTGCGATTTGCAGAGCCATT |
